# MultiElec: A MATLAB Based Application for MEA Data Analysis

**DOI:** 10.1371/journal.pone.0129389

**Published:** 2015-06-15

**Authors:** Vassilis Georgiadis, Anastasis Stephanou, Paul A. Townsend, Thomas R. Jackson

**Affiliations:** 1 Institute of Child Health, University College London, London, United Kingdom; 2 Institute of Cancer Sciences, University of Manchester, Manchester, United Kingdom; 3 Manchester Centre for Cellular Metabolism, Manchester Cancer Research Centre, Faculty of Medical and Human Sciences, University of Manchester, Manchester, United Kingdom; Dalhousie University, CANADA

## Abstract

We present MultiElec, an open source MATLAB based application for data analysis of microelectrode array (MEA) recordings. MultiElec displays an extremely user-friendly graphic user interface (GUI) that allows the simultaneous display and analysis of voltage traces for 60 electrodes and includes functions for activation-time determination, the production of activation-time heat maps with activation time and isoline display. Furthermore, local conduction velocities are semi-automatically calculated along with their corresponding vector plots. MultiElec allows ad hoc signal suppression, enabling the user to easily and efficiently handle signal artefacts and for incomplete data sets to be analysed. Voltage traces and heat maps can be simply exported for figure production and presentation. In addition, our platform is able to produce 3D videos of signal progression over all 60 electrodes. Functions are controlled entirely by a single GUI with no need for command line input or any understanding of MATLAB code. MultiElec is open source under the terms of the GNU General Public License as published by the Free Software Foundation, version 3. Both the program and source code are available to download from http://www.cancer.manchester.ac.uk/MultiElec/.

## Introduction

The use of microelectrode array (MEA) systems has become increasingly common in both neurological and cardiological sciences [1,2]. MEAs allow stimulation and recording of bioelectricity with high spatial and temporal resolution *in vitro* (cell and tissue culture) [3]. Signal recorded by MEAs are the extracellular field potentials (FPs), allowing non-disruptive measurements to be collected at a cell population level [4].

Many *in vitro* preparations have been analysed using MEAs such as; cardiomyocyte monolayer cultures [5], including those differentiated from induced pluripotent stem (iPS) cells [6–12]; cardiac slices and cardiac sheets generated *in vitro* [13]; cortical cultures using cultures derived from dissociated cells [14,15] and intact cortical slices [16,17]; spinal cord cultures [18]; and intact retina [19]. Studies range from recapitulating disease characteristics *in vitro* using cardio myocytes produced from the differentiation of human iPS cells carrying specific mutations [8,11] to the production of neuroregeneration models and the subsequent screening and evaluation of neuroregenerative promoting compounds [20]. The biological application of MEAs for basic research and drug discovery has been reviewed extensively [1,21].

MEA recordings produce large amounts of data, which are traditionally analysed manually, however, such analysis is labour intensive, slow and user-dependent [22]. Commercial software are available, however they do suffer certain drawbacks. For example Cardio2D requires data to be in the native format of its own specialised recording software, limiting its use [22]. Furthermore, the analysis of the data is somewhat opaque when using commercial software. Several laboratories have utilized custom MATLAB scripts to analyse MEA data [6,19,23]. However, not all users of MEA are fluent in MATLAB code. Multi Channel Systems (MCS) have produced open source MATLAB scripts for the analysis of MEA data in the form of MEA Tools [24], yet some understanding of the use of and access to MATLAB is still required for the implementation of these scripts. Furthermore, MEA tools have not been updated for some time. Other custom MATLAB scripts and toolboxes have also been produced; importantly, these are not open source. Recently, open source software CardioMDA was released to analyse of field potential duration, however, it does not analyse signal propagation. It is clear that the progression in MEA technology is quickly outstripping our ability to analyse the data.

Here we present MultiElec, a user-friendly application specifically designed for the analysis of *in vitro* MEA data with a particular focus on the semi-automatic measuring of conduction velocities. The ability to determine conduction velocity is a unique advantage of using MEA recordings over traditional electrophysiological methods. To calculate conduction velocities methods previously described by Bayly et al [25], were utilised and integrated into a graphic user interface (GUI). Bayly’s method involves the fitting of polynomial models to activation time data and solving the total derivatives to obtain velocity vectors. This method is robust to missing data and the models can be assessed for goodness of fit to calculated activation times and thus the quality of the subsequent conduction velocity calculations can be estimated.

Our software was developed with a focus on cardiomyocyte preparations but this could be utilised for other *in vitro* preparations using the MEA system.

To test MultiElec we used it to assess the response of neonatal rat cardiomyocytes to the addition of adrenaline to culture media with that hypothesis that this would cause an increase in beta rate but not affect the conduction velocity of the field potential.

## Materials and Methods

### Ethics Statement

This study was performed in accordance with the United Kingdom Home Office Animals (Scientific Procedures) Act 1986. The animals were euthanized by cervical dislocation (UK approved Schedule One Method for rodents). Home office UK project licence: PPL 70/6935 13-Jul-2009.

### Neonatal rat ventricular myocyte culture

Neonatal rat ventricular cardiomyocytes were isolated from the hearts of 1–2-day-old Sprague–Dawley rats. Hearts were removed and placed in oxygenated ADS buffer (116 mM NaCl, 5·4 mM KCl, 20 mM HEPES, 0·8 mM NaH_2_PO_4_, 405·7 μM MgSO_4_ and 5·5 mM glucose, pH 7·35). Heart tissue was digested in 10 ml oxygenated ADS buffer supplemented with 0·1% collagenase Type II (Worthington) and 0·025% pancreatin for 15 min, and the liberated cells were pelleted at 1300 rpm for 5 min and resuspended in fetal bovine serum (FBS) (Life Tech). This digestion procedure was repeated eight times, after which the cells were plated at 37°C for 1 h to allow the adherence of fibroblasts. Cardiomyocytes were plated at a density of 2·5×10^5^/ml in DMEM (Life Tech) with 40 units/ml penicillin (Gibco), 40 μg/ml streptomycin and 15% FBS. Cells were allowed to attach to the plates overnight, and the medium was replaced with DMEM containing 1% FBS. All reagents were obtained from Sigma-Aldrich unless otherwise stated.

### Microelectrode array (MEA) extracellular recordings

Cardiomyocytes were seeded at a density of 2x10^5^ in the centre of MEA glass arrays coated with a 10 μl fibronectin (20 μg/ml) (MEA specification: Electrode material: TiN, glass substrate, 8x8 60-electrode configuration, planar 30 μm diameter electrodes, 200 μm interelectrode distance. SiN isolator) (Multi Channel Systems MCS, GmbH). Media (DMEM, 1% FBS) was replenished every 24 hours. After 6 days in culture, recordings of spontaneously contracting cultures were taken at 20kHz sampling rate, using an MEA 1060 amplifier with a gain of 1100 and frequenzy bandwidth is 1Hz—3 kHz. The recording medium was the same as the plating medium. For adrenergic stimulation, 10 μM adrenaline (Sigma, Cat No: E4250) was added to the media during recording and the culture was gently resuspended. Raw data were recorded on the MC Rack software (Multi Channel Systems MCS GmbH).

### Software engineering and system requirements

MultiElec, was programmed in MATLAB R2012a (The Math Works Inc., Natick, Massachusetts, United States) and compiled using the MATLAB compiler (The Math Works Inc., Natick, Massachusetts, United States) to create a standalone executable. Thus, MultiElec is available as an executable that can be run independently of MATLAB installation and without a MATLAB licence. The software has been tested on Windows 7 Enterprise, Windows 7 and Windows 8 with a 64-bit-operating system.

### Data format

Signals are recorded using MC_Rack (Multi Channel Systems, Reutlingen, Germany), which produces data files in the mcd format. MC_DataTool (Multi Channel Systems, Reutlingen, Germany) software can be used to simply covert these files into an ASCII format, which can then be loaded and utilised by MultiElec.

### Program work flow and graphic user interface

**Fig 1:**
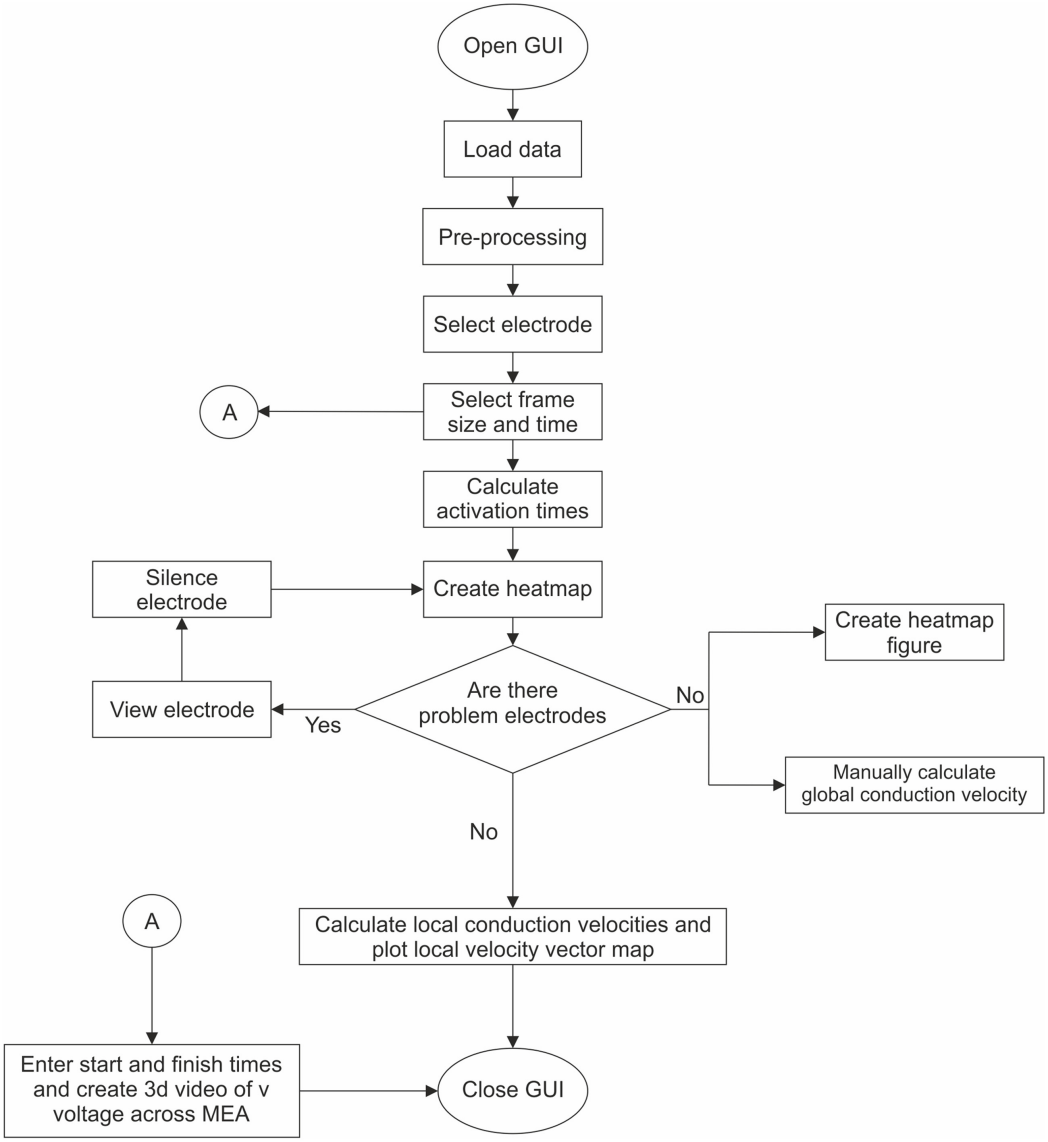
Flow chart of MultiElec work flow. On execution of the program the GUI is opened. The user can then select data to be loaded. Once the data is loaded the program will pre-process the data (filters noise). The user can then select an electrode and focus on a particular signal event. Once a signal event is in view the user can request that activation times are calculated and activation time heat maps are produced. Using these initial heat maps the user can silence problem electrodes and repeat the process until all problem electrodes are silenced. Once appropriate electrodes are silenced conduction, velocities and subsequent velocity vector maps can be produced. At various points during this process figures can be produced and saved for further reference or publication.

**Fig 2:**
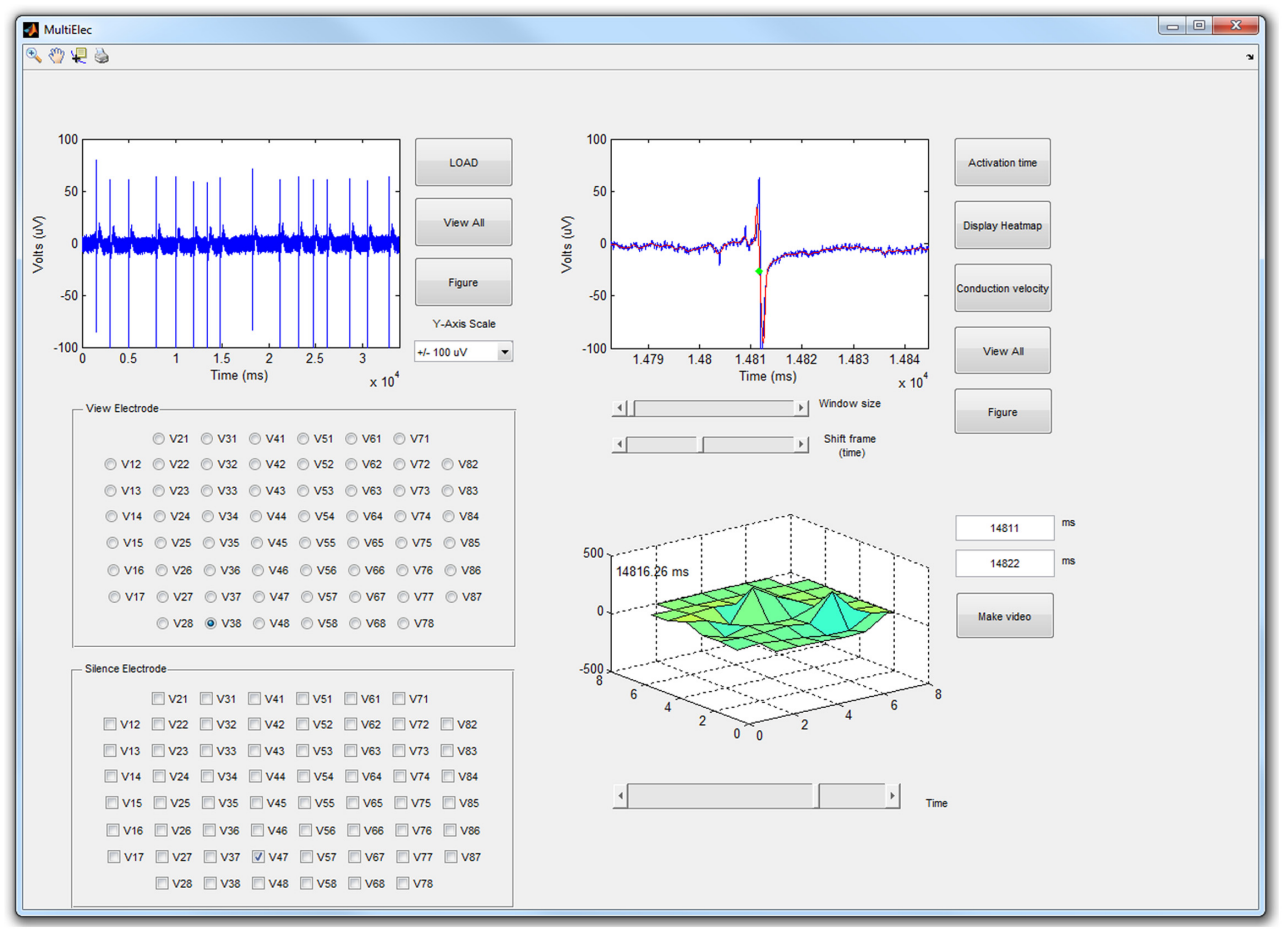
Screen shot of MultiElecs’ GUI. The ‘view electrode’ panel allows the user to select individual electrode to be viewed. The ‘silence electrode’ panel allows the removal of selected electrodes from analysis. The top left plot shows the entire recording for the selected electrode. The top right plot allows the user to zoom in at a chosen time for analysis of the field potential (green dot marks the calculated activation time). The bottom right plot allows the user to view the voltage of the entire set of electrodes change with time.

The flow chart of the MultiElec work flow is shown in Fig 1. The application was designed to be user friendly and to be accessible to users with no knowledge of MATLAB coding. To this end the application can be controlled entirely via a simple graphic user interface (GUI) (Fig 2). From this GUI, data can be loaded, analysed and figures produced without the need of command line input. Once the data is loaded the user can select an electrode to use for signal analysis. Window size and position sliders allow the user to easily focus on a wave front. Activation times and conduction velocities can then be calculated and these used to produce heat maps, and conduction velocity vector maps. Various options are available for figure production as the user progresses through the data analysis.

### Visualisation of signal

**Fig 3:**
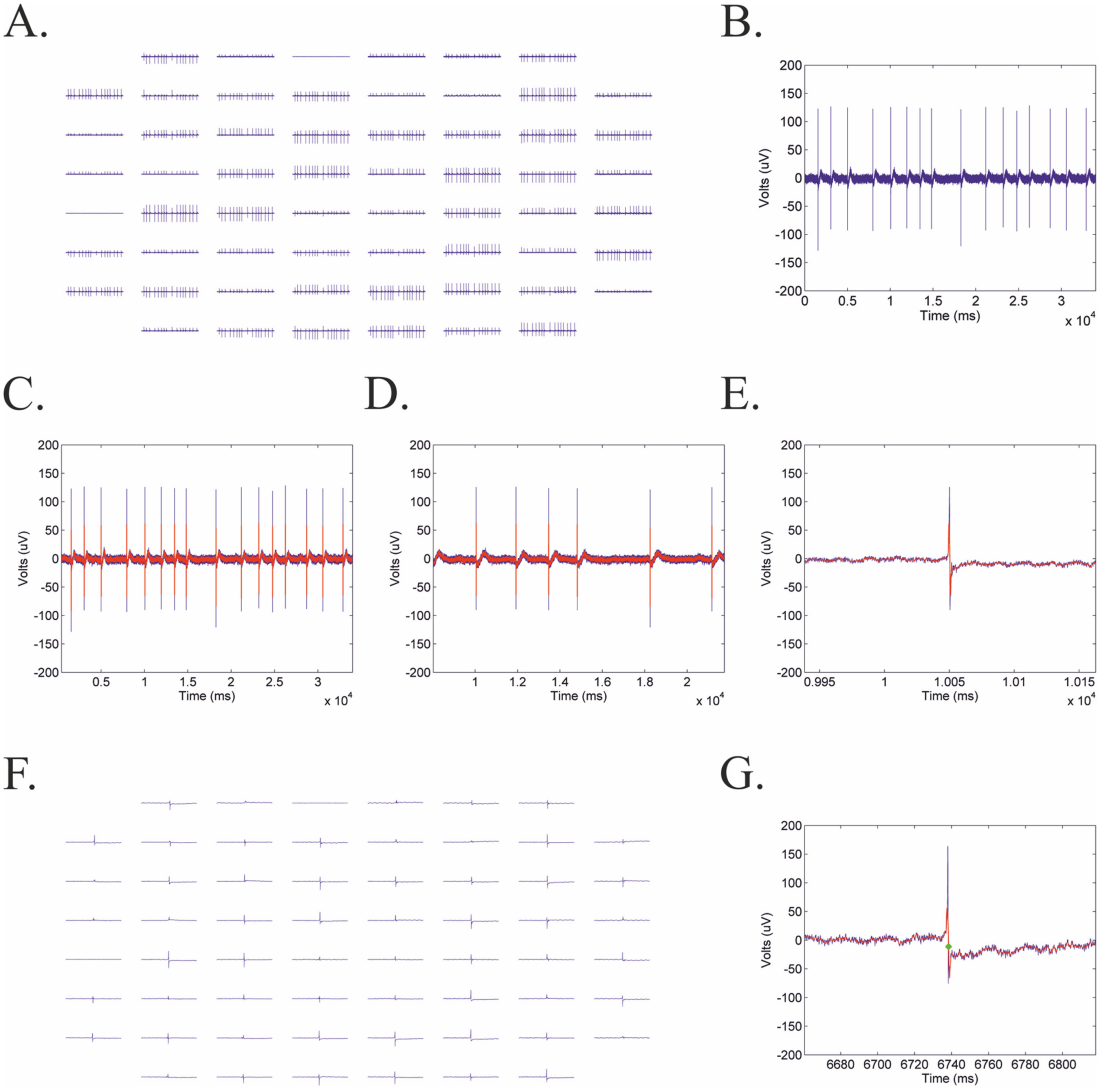
Examples of voltage plots exported form MultiElec. A) Full length recording for all sixty electrodes. B) A full length recordings for a single electrode. C-E) Sliders enable the user to zoom and focus on a single event/wave front. F) Shows all sixty electrodes zoomed in on a single event. G) Shows single event with calculated activation time marked in green. Smoothed signal superimposed in red.

MultiElec allows several different views of the voltage signal. The GUI allows the user to view the full recording for all electrodes at once (Fig 3A) or to select a single electrode (Fig 3B). Using sliders to adjust the scale of the view (window size) window and when in the recording to view (time), the user is able to zoom and focus on a single signal event/wave front (Fig 3C–3E). This window is automatically allied to all electrodes (Fig 3F).

### Activation time determination and heat maps

As part of the pre-processing of the data the electrode signals are smoothed by the use of a Savituzky-Golay smoothing filter using a polynomial of order 3 and a “half width” of 50 data points (frame size of 101 (2.04ms)). The properties and applications of these filters have been reviewed elsewhere [26–28]. The normalised cut off frequency *f*_*c*_ = *w*_*c*_ */π* can be predicted by the following equation

$$f_{c}=\frac{N+1}{3.2M-4.6}$$ (1)

where N is the polynomial order and M is the half width [29]. Thus in this case f_c_ = 0.0346. With a sampling frequency at 20 kHz the resulting cut of frequency is approximately 0.7 kHz.

The activation time at each electrode is determined as the time of the steepest negative gradient (dv/dT_min_), a common method for the detection of cadiological activation times [6,19,23,30–33]. The numerical gradient was determined using the MATLAB “gradient” function. For reference the activation time is marked on the recoding of the selected plot (Fig 3G). This method yielded a specificity of 86.27%, a sensitivity of 96.27% and a false positive rate of 13.72%. False positives are, however, easily recognised on heatmaps and electrodes can subsequently be manually silenced by the user.

**Fig 4:**
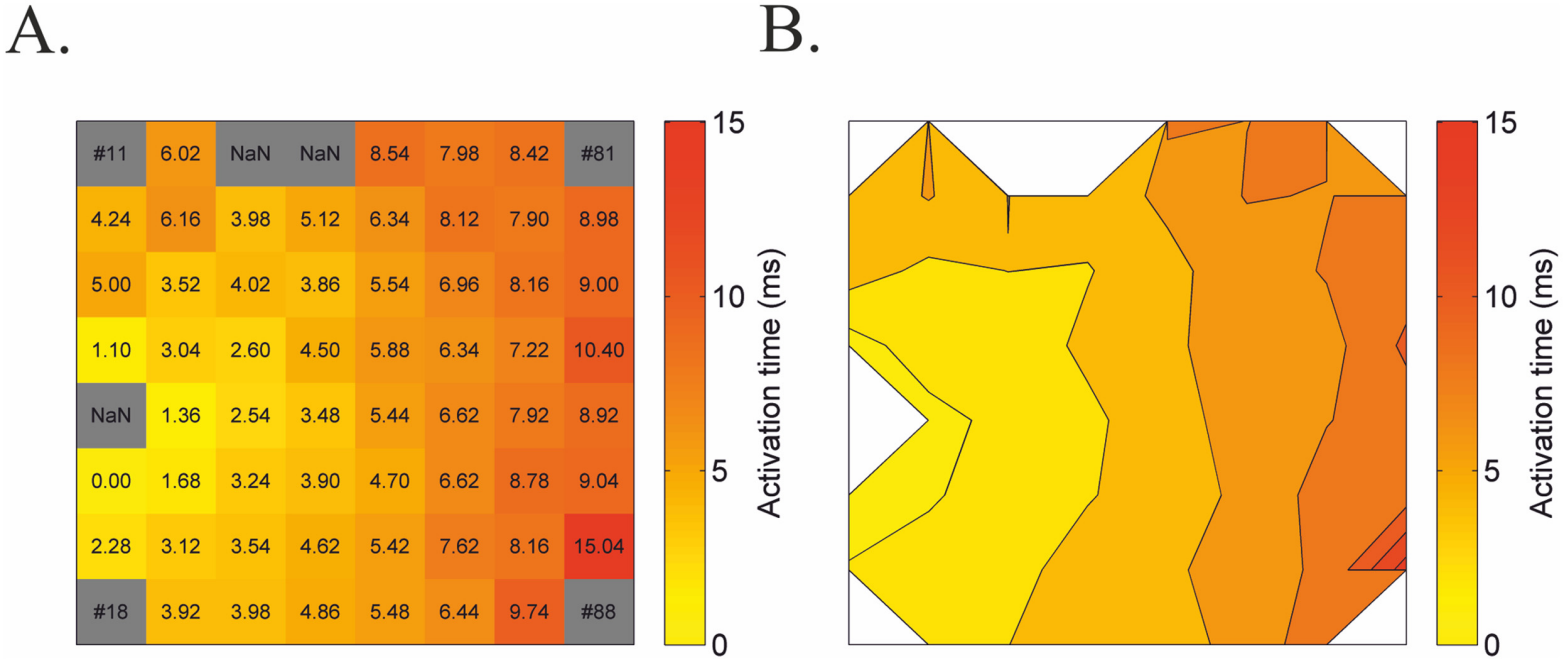
Examples of heat maps produced by MultiElec. A) Heat map with activation times at each electrode. B) Heat map with isolines.

MultiElec utilises the calculated activation times to produce two different types of heat maps. The first displays a grid of activation times at each electrode and is colour-coded accordingly. This is useful for visualising the exact activation times for each electrode and hence the visualisation of ‘problem electrodes’/artefacts (Fig 4A). These activation times can also be used for subsequent manual conduction velocity calculations. In addition, a second plot displays a heat map that includes isolines of the activation times (Fig 4B). This allows a visual interpretation of changes in signal velocity with signal progression. As such, far spaced isolines indicate a fast moving signal whereas closer isolines indicate a slower moving signal.

### Conduction velocities

Conduction velocities are traditionally calculated from activation time heat maps using finite difference methods. Briefly, the direction of signal propagation is determined manually using activation time heat maps (this can be somewhat subjective), and then the speed is calculated as the difference in activation time between two electrodes along the decided direction of signal propagation divided by the distance between the two electrodes. For such approaches to be valid local activation times have to be precisely determined and hence depend on high temporal and spatial resolution. In addition, the direction of propagation should be accurately known and herein lays the problem. If the direction of signal propagation is not approximately parallel to the line connecting the two electrodes but instead is almost perpendicular then this method will result in large overestimates in velocity [34].

MultiElec uses an automated method based on that previously described by Bayly et al. [34]. It relies on the fitting of polynomial surfaces *T*(*x*, *y*) to observed electrode activity data (*x*, *y*, *t*), where *x* and *y* are electrode co-ordinates and *t* is the calculated activation time. The polynomial surface was defined as *t*_*i*_ = *T*(*x*_*i*_, *y*_*i*_)

$$T\left(x,y\right)=ax^{2}+by^{2}+cxy+dx+ey+f$$ (2)

**Fig 5:**
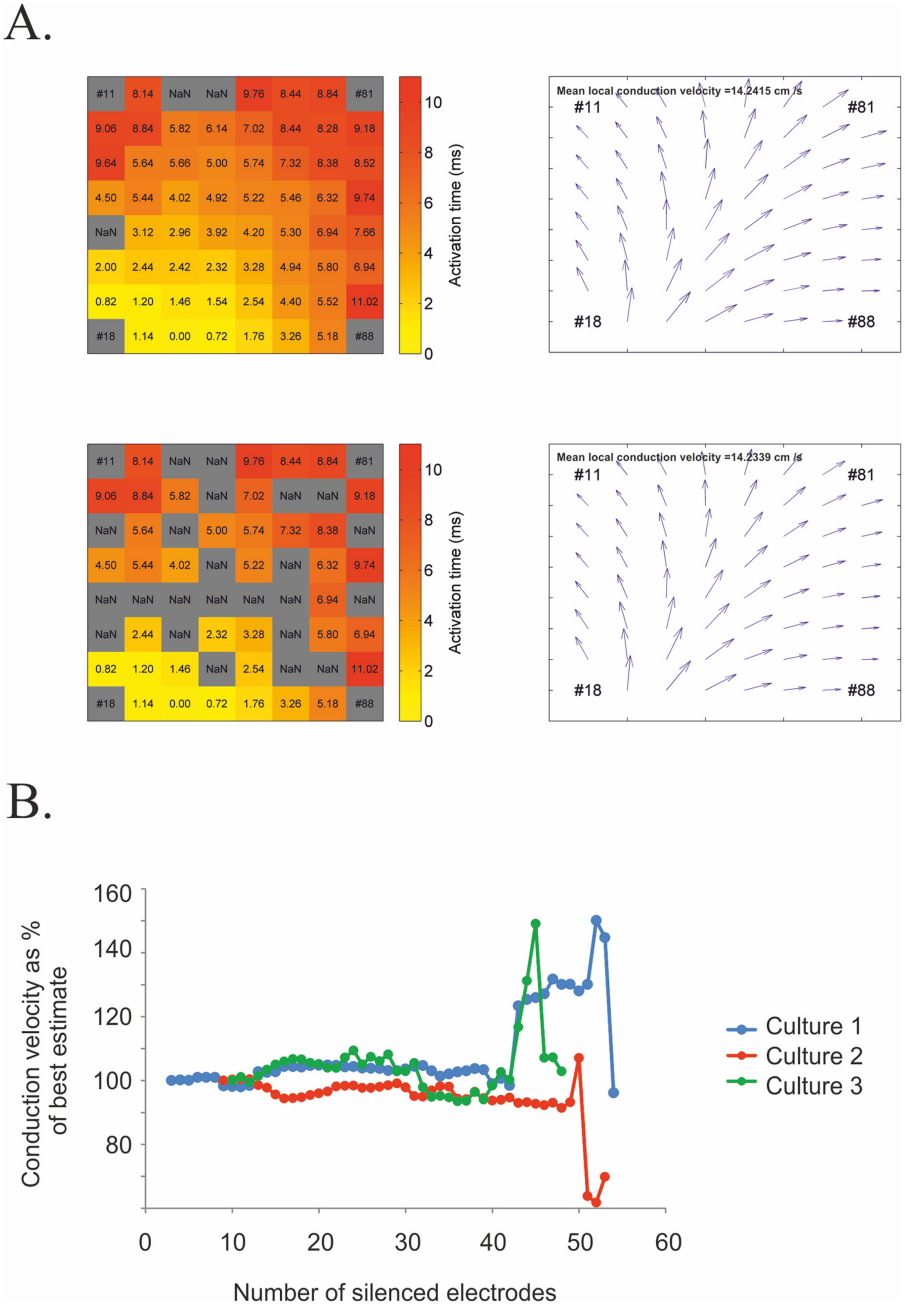
Comparison of vector maps with complete and incomplete data. A-B) Heat map of complete (top) and incomplete (bottom) data set and corresponding vector field and mean local conduction velocities. B) Plot of estimated mean local conduction velocity as a percent of the best estimate (using all available errors) against number of silent electrodes. Error is relatively low and stable for low numbers of silenced electrodes but dramatically increases >35 electrodes are silenced. Three independent experiments are shown.

The polynomial is fitted using least-square algorithms using all available data points on the MEA recording (S1A Fig). Bayly et al. demonstrated that the velocity vector at each electrode are then defined as

$$V=\left[\begin{array}{c} \frac{dx}{dt}\\ \frac{dy}{dt} \end{array}\right]=\left[\begin{array}{c} \frac{T_{x}}{T_{x}^{2}+T_{y}^{2}}\\ \frac{T_{y}}{T_{x}^{2}+T_{y}^{2}} \end{array}\right]$$ (3)

where *T*_*x*_ = ∂*T /* ∂*x* and*T*_*y*_ = ∂*T /* ∂*y* [34]. These vectors are used to produce vector field plots and calculate mean local conduction velocities (Fig 5A). Fitting polynomials to estimate local conduction velocities has several advantages over finite different methods. Firstly, polynomial fitting is robust to missing data. If data is missing using finite different algorithms then the algorithm would have to be adapted or interpolation used. This is not necessary for polynomial fitting. Further, polynomial fitting is also intrinsically smoothing whereas finite different methods tend to amplify noise [34]. To analyse this models robustness to missing data an increasing number of electrodes were randomly silenced and the subsequent change in mean local conduction velocity assessed (Fig 5). As can be seen from Fig 5 the model can tolerate a large number of silenced electrodes without a substantial deviation from the best estimate (using maximum number of available electrodes) of the mean conduction velocity. As well as the mean conduction velocities remaining comparable with silenced electrodes the vector field’s shame also remains comparable. In the data sets analysed substantial changes in the mean conduction velocity appear once the total number of used electrodes decreases below 20. In addition, when using polynomial fitting subjectivity in deciding direction of propagation is removed, repeatability is increased and the quality of velocity vectors can be assessed by using the residual error of the fit (S1B Fig). A good fit will have residuals that are approximately normally distributed around zero and randomly distributed in terms of predicated activation.

### Robustness to noise

**Fig 6:**
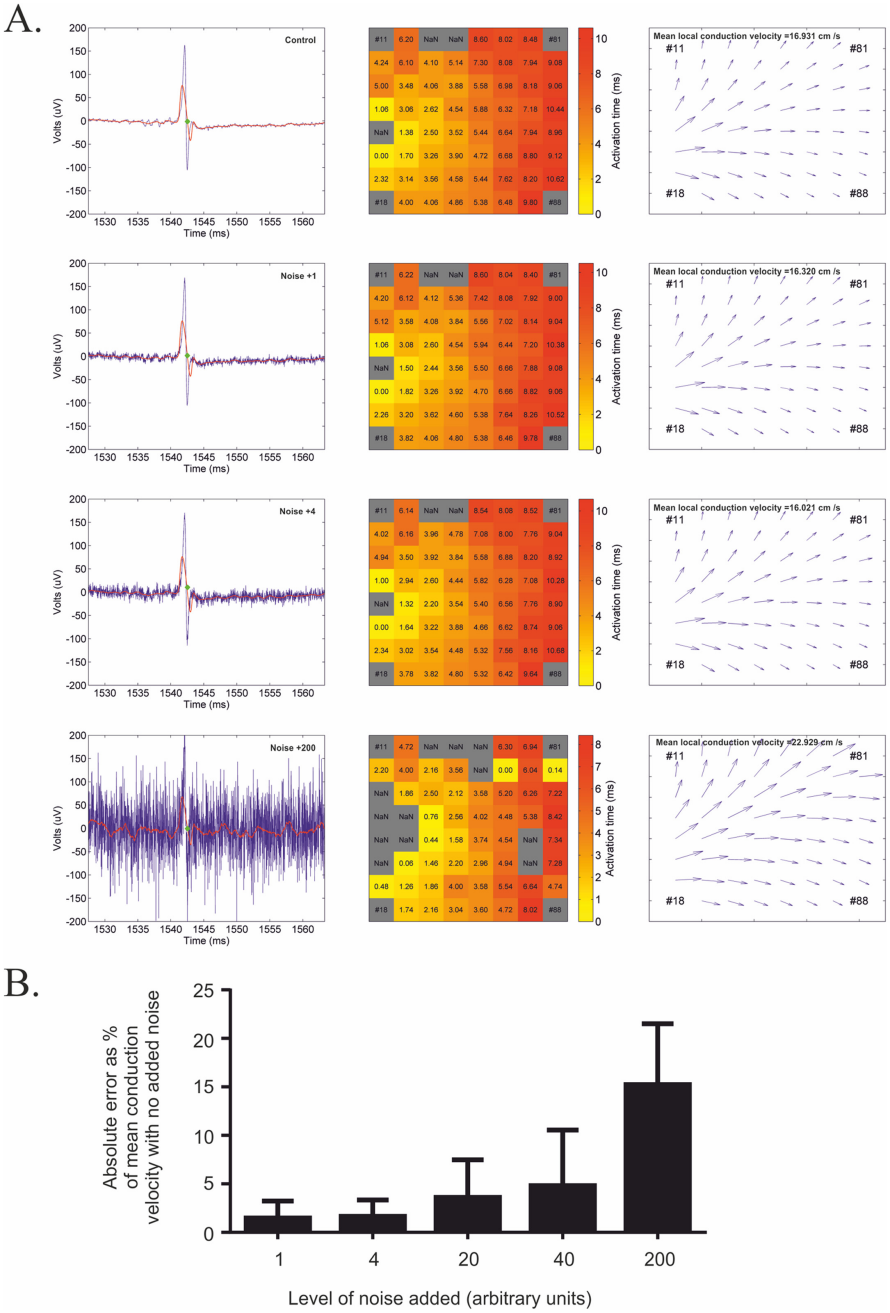
Assessment of the effect of noise on predicted conduction velocity. A) FP plots, heat maps and conduction velocity vector maps are shown for a data set with increasing amounts of white noise added. B) Plots of absolute error with indicated noise level when compared to no added noise. Error bars represent SEM, n = 5.

The ability of this detection system to tolerate noise was further assessed by adding white noise to data sets and assessing changes in calculated conduction velocity (Fig 6). This analysis demonstrated that both the detection method and subsequent conduction velocity calculations determination was robust to relatively high levels of noise (Fig 6A and 6B, noise +4) substantially higher than those expected to be seen in MEA recordings. Surprisingly, it was still possible to detect activation times with extremely high levels of noise (Fig 6A, noise +200), however, predictably the subsequent error in activation times and mean local conduction velocities becomes unacceptably high (Fig 6B).

### 3D videos of signal progression

**Fig 7:**
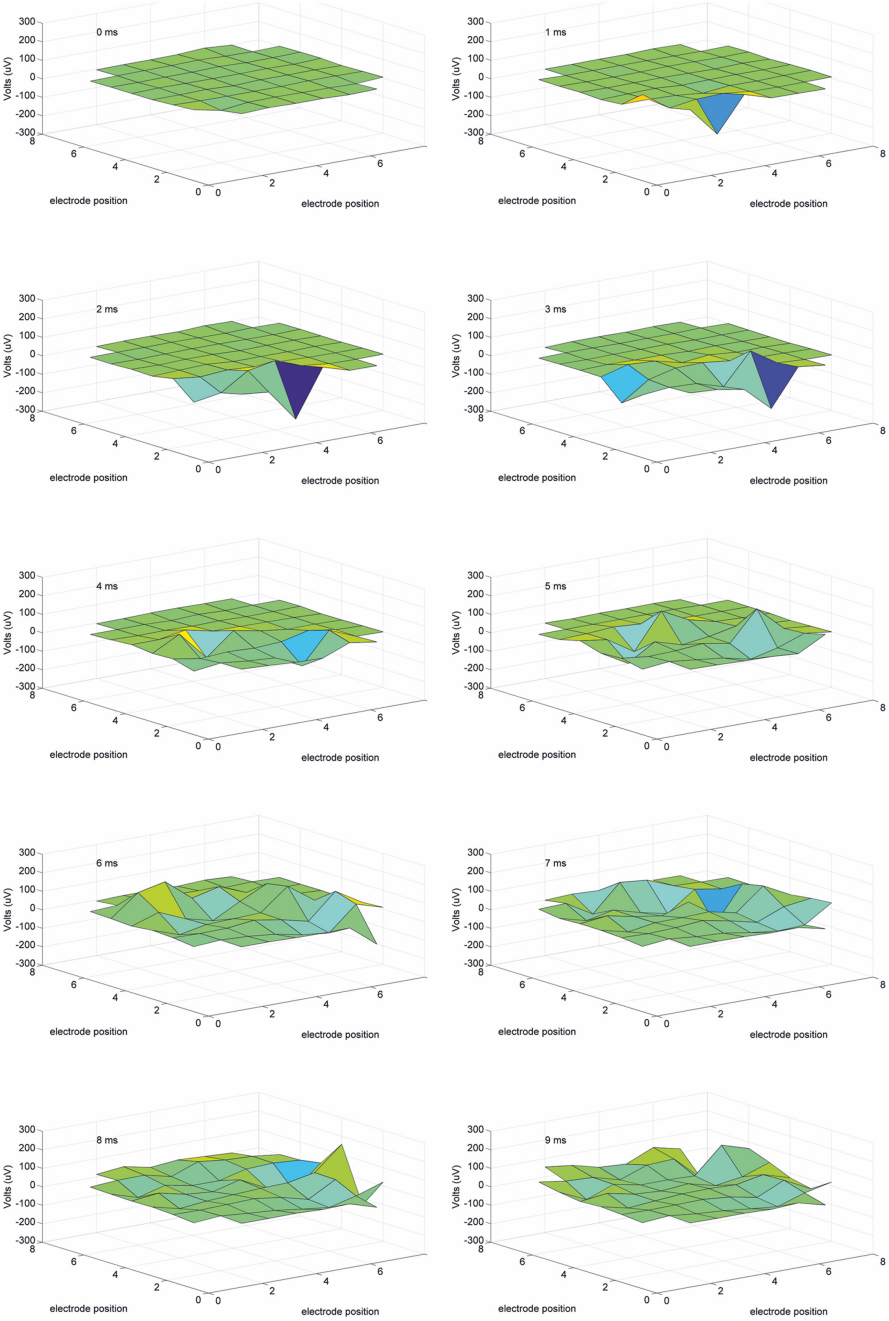
Still frames of a 3D movie recording showing signal progression across electrodes. 9ms recording shows the signal initiation at the bottom left corner of the array and progress outwards.

MultiElec offers a unique 3D movie representation of the changes in voltage across all electrodes over time (S2 and S3 Figs). Snap shots of a movie showing signal progression are shown in Fig 7. The movies allow a more intuitive view of the data than an array of separate voltage traces and allows the data that is normally lost when viewing heat maps (such as magnitude of voltage change) to be viewed with signal progression. Further, this view displays the raw data, thereby removing error that might be introduced from defining activation times by a mathematical property of the trace. We feel that the more complete and raw view of the 3D movies complements the more processed interpretation of data via the determination of activation times, heat maps and conduction velocities.

### Limitations

We have emphasised here that the main strength of MultiElec is that it provides a user friendly GUI and that no special skills or knowledge are required to use it. With this property comes its main weakness, the very nature of having an application such as MultiElec instead of individual MATLAB scripts is that it has a lack of flexibility. Unlike individual scripts MultiElec is not easily manipulated, and those that can, will likely prefer to create their own custom MATLAB scripts or adapt published scripts to analyse MEA data. MultiElec does not currently have a spike sorting function and is thus not suitable for the analysis of the processing of neural data.

## Results and Discussion

MEAs allow the recording of extracellular field potentials allowing the bioelectrical properties of cells to be measured non-invasively at a population level. The production of MEA recordings are relatively simple and do not require such specialised skills necessary for more traditional methods such as patch clamping. In addition, MEA arrays allow an extra dimension of information such as FP progression through a cell population to be measured. However, the subsequent analysis of the data is complex and the development of suitable analytical software has been tardy. Thus, large amounts of data produced by this technique are not always used to their full potential. Manual analysis of such data can be labour intensive causing a bottleneck in what would otherwise be a high throughput approach. In particular the opportunity provided by multiple electrodes to calculate conduction velocities appears to be underutilised. We have developed MultiElec to assist in the analysis of MEA data and here we demonstrate its use. The intuitive GUI makes the program simple to use and will allow a more systematic and time efficient analysis of MEA data, specifically with the calculation of local conduction velocities.

**Fig 8:**
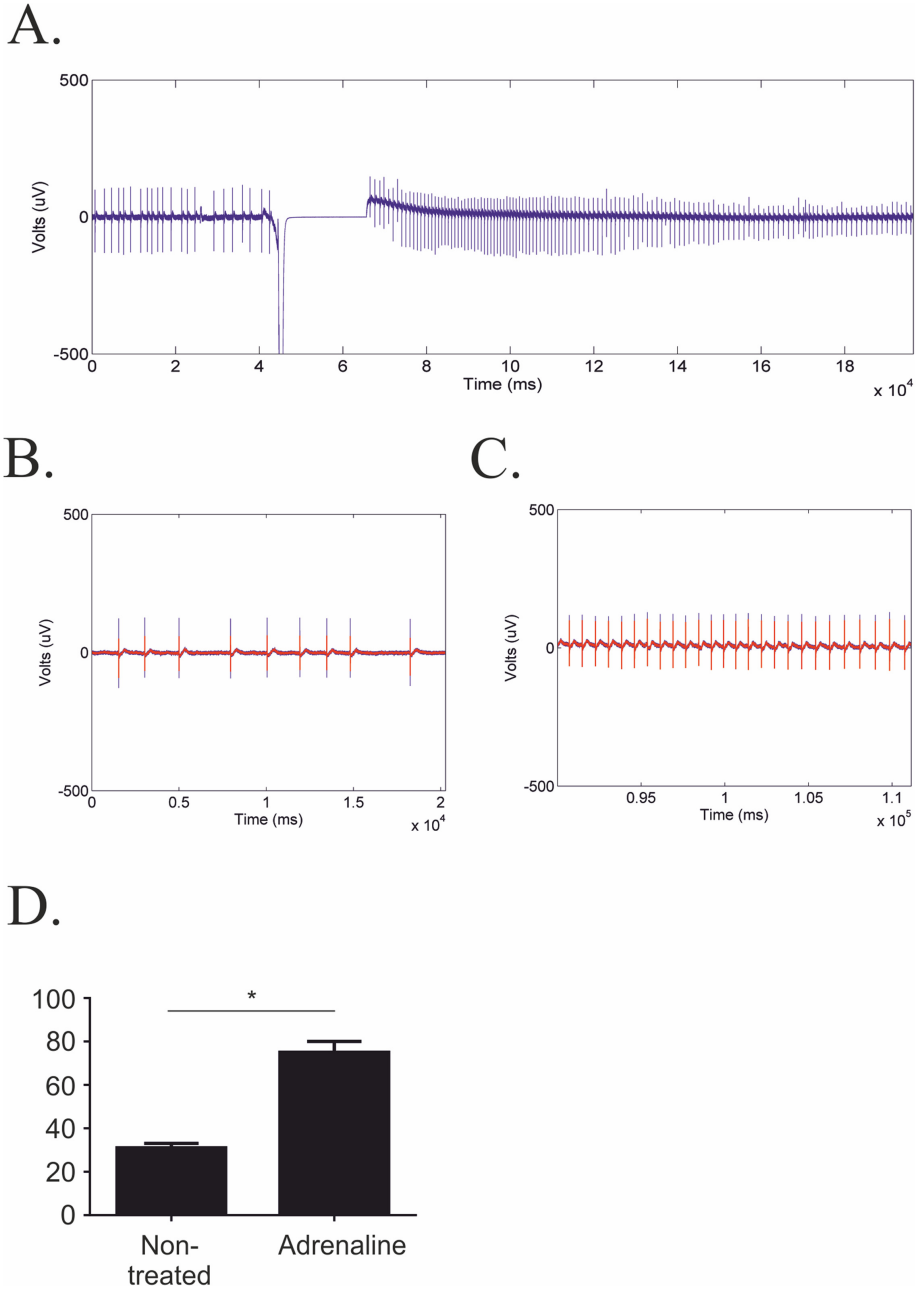
Comparison of beat frequency before and after adrenaline treatment. A) Full length recording. B) Zoomed recording before addition of adrenaline. C) Zoomed recording after adrenaline. D) Quantitative comparison of beats per min, data represents mean ± SEM. A paired students t-test was performed and the difference found to be significant, *p<0.05, n = 4.

By way of testing the MultiElec program we assessed neonatal rat cardiomyocytes grown on MEA culture slides for 6 days before the extracellular field potentials were then recorded immediately before and after the addition of adrenaline to the culture media. The raw data from the MEA recordings was then analysed using MultiElec. A continuous recording was made during the process of adding the adrenaline which involved the addition of 10 M of adrenaline to the MEA media, followed by gentle resuspension of the media and this process can be detected by a period of disrupted signal which was used to mark the addition of adrenaline (Fig 8A). Magnified images before and after the addition of adrenaline (Fig 8B and 8C) allowed the beats per minute to be quantified and as expected the addition of adrenaline to cardiomyocytes significantly (p>0.05, paired t-test, n = 4) increased the beat frequency (Fig 8D).

**Fig 9:**
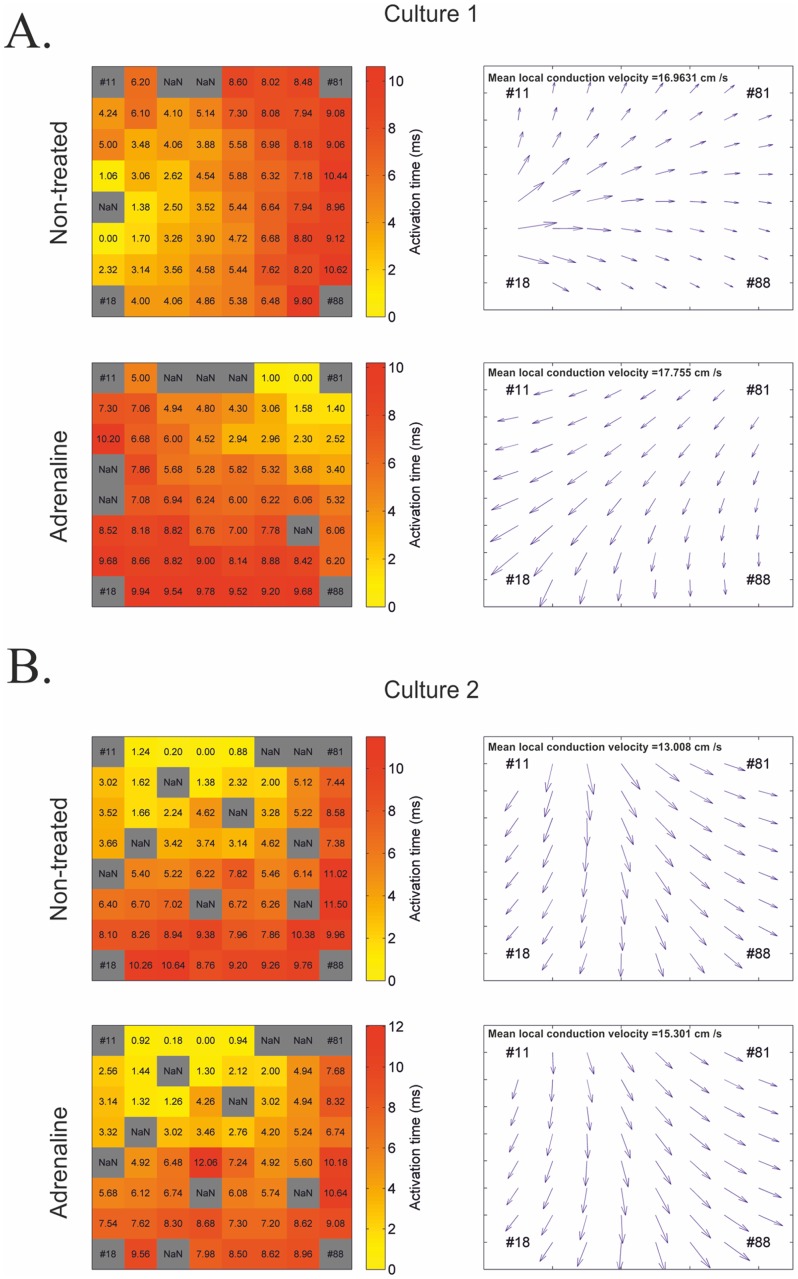
Comparison of conduction velocities before and after adrenaline treatment. A and B) Heat maps with activation times and conduction velocity vector plots for non-treated and adrenaline-treated cultures, respectively. A and B from two independent experiments.

MultiElec was subsequently used to assess the signal propagation of the FPs. Fig 8 shows representative heat maps and conduction velocity vector maps before and after the addition of adrenaline. We found that the mean local conduction velocities did not substantially change with the addition of adrenaline. However, due to a lack of primary material we were unable to analyse this data with statistical vigour. We have instead provided 2 representative recordings in Fig 9 and a third in S4 Fig. In addition we observed changes in the orientation of signal progression, examples of which can be seen in Fig 9A and supplementary Fig 2. The simplest explanation for this is that the physical disruption of changing the media provided an opportunity for an alternative pacemaker to replace the original pacemaker and pace the syncytium. Alternatively, it is possible that the increased beat rate has resulted in an increased refractory period and pressure to "fit" between the refractory periods has resulted in a change in pacemaker and signal progression orientation.

Movies of signal progression were produced and representative movies can be found in the supplementary material (S2 and S3 Figs). In these movies a change in orientation in the signal propagation is observed but the speed of signal propagation and magnitude of FPs remain comparable.

## Conclusions

Here we present MultiElec, an open source program designed to make the analysis of MEA recordings and the determination of conduction velocities more efficient and systematic. The program was successfully used to analyse neonatal rat cardiomyocytes treated with adrenaline.

## Supporting Information

S1 Fig Examples of data fitting. Upper panel shows model fit (mesh) to raw data (circles). Lower panel shows predicted values of model plotted against residuals.
(TIF)

S2 Fig 3D video of field potential progression for control cells. (AVI)

S3 Fig 3D video of field potential progression for adrenaline treated cells. (AVI)

S4 Fig Comparison of conduction velocities before and after adrenaline treatment. Heatmap with activation times and conduction velocity vector plot for non-treated and adrenaline-treated cultures, respectively.
(TIF)
